# Camouflage Orthodontic Treatment With Miniscrew Anchorage for Facial Profile Enhancement in a Severe Hyperdivergent Skeletal Class II Protrusion Case

**DOI:** 10.1155/crid/6498137

**Published:** 2026-02-23

**Authors:** Lijie Zhang, Jiayuan Zhao, Tianyu Fu, Nasser Saeed Bahaj, Qian Ye, Xiaoli An

**Affiliations:** ^1^ School/Hospital of Stomatology, Lanzhou University, Lanzhou, 730000, China, lzu.edu.cn

## Abstract

Treatment of severe hyperdivergent skeletal class II protrusion in adult patients has always been a significant clinical challenge for orthodontists. For the optimal enhancement of facial esthetics, orthodontic‐orthognathic treatment may represent the most appropriate treatment option. However, this case report presents the successful camouflage orthodontic treatment of a 25‐year‐old female patient who had a skeletal Class II malocclusion with a convex profile, retrusive mandible, a slight high plane angle of the mandible, individual anterior crossbite, posterior scissor bite, and lip muscle hypertonicity. The treatment plan included extraction of all first premolars and maximum anterior retraction with miniscrew anchorage for optimal profile. The active treatment phase lasted 36 months. Posttreatment evaluation revealed significant improvement in facial profile and optimal occlusal relationship, which demonstrated clinical stability through a 1‐year retention period. This case illustrates that camouflage orthodontic treatment is an effective therapeutic option for adult patients with severe skeletal Class II malocclusion, achieving satisfactory facial esthetics, functional occlusion, and long‐term stability. Miniscrew anchorage additionally simplifies the treatment protocol while avoiding the inconvenience and discomfort associated with conventional anchorage devices.

## 1. Introduction

Class II malocclusion is a prevalent dentofacial deformity in clinical practice, with a systematic review reporting a global prevalence of 24.7% [1]. The etiology of skeletal Class II malocclusion is multifactorial, involving genetic predisposition, aberrant craniofacial growth, and environmental influences [2–4]. Skeletal Class II malocclusion is clinically characterized by sagittal discrepancies in maxillomandibular relationships, specifically presenting as maxillary prognathism, mandibular retrognathism, or a combination of both [5]. In cephalometric analysis, the ANB angle exceeding 4.5° is pathognomonic for skeletal Class II malocclusion [6]. However, definitive diagnosis requires correlation with clinical examination and dental model analysis. Many patients with skeletal Class II malocclusion often exhibit a convex facial profile, which is also the primary reason they sought orthodontic treatment [7].

The achievement of facial esthetics is one of the primary objectives in orthodontic treatment. For optimal esthetic outcomes, combined orthodontic‐orthognathic therapy is often the preferred approach for patients with severe skeletal malocclusion [8–10]. However, some patients may opt for orthodontic camouflage treatment as a nonsurgical alternative, which often needs premolar extraction and maximum anchorage to achieve treatment objectives [11–14]. Existing studies have confirmed that miniscrew anchorage demonstrates superior clinical efficacy compared to conventional orthodontic appliances in orthodontic treatment [15, 16].

This case report describes the camouflage treatment of skeletal Class II malocclusion with a convex profile, retrusive mandible, a slight high plane angle of the mandible, individual anterior crossbite, posterior scissor bite, and lip muscle hypertonicity. We made the treatment plan to extract four first premolars and use miniscrew anchorage for space closure, anterior teeth retraction, and intrusion. The treatment effectively provided profile improvement.

## 2. Case Report

### 2.1. Diagnosis and Etiology

This patient was a 25‐year‐old Chinese female with the chief complaints of a protrusive mouth and malaligned teeth. She reported no contributing medical history and denied any family history of malocclusion and deleterious oral habits. Her periodontal tissue and temporomandibular joints were asymptomatic.

**Figure 1:**
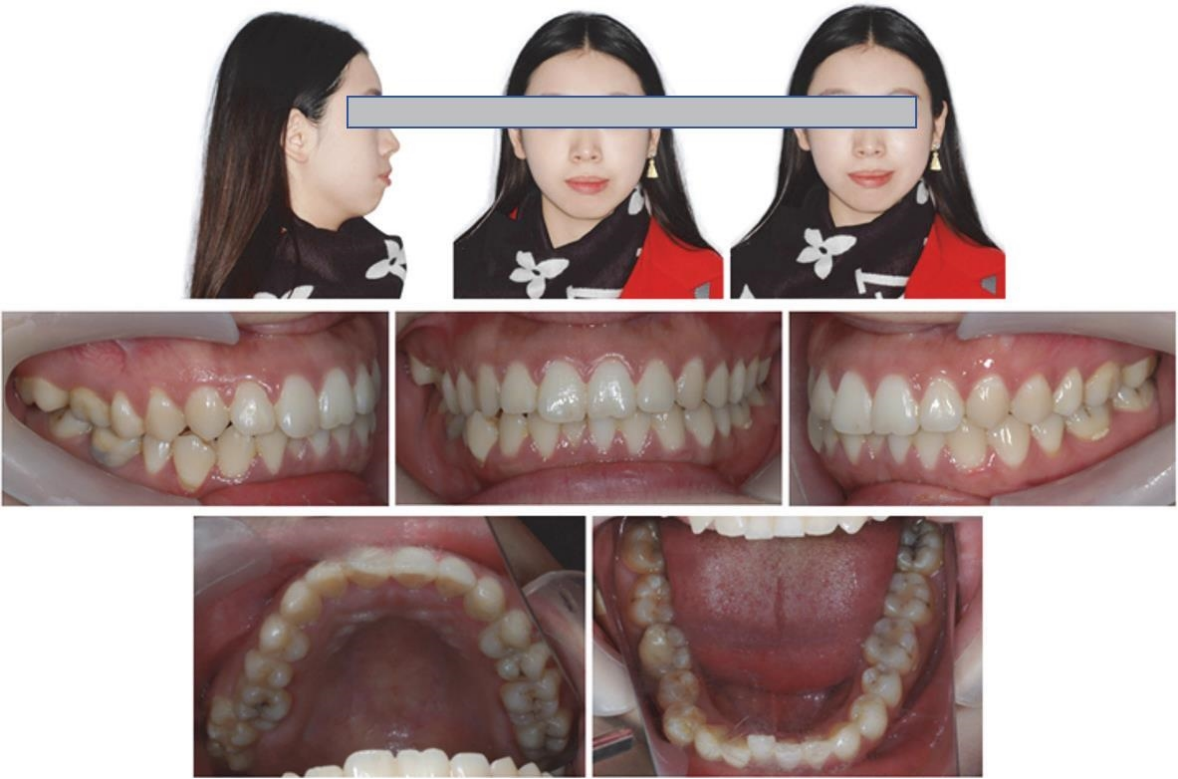
Pretreatment facial and intraoral photographs.

The pretreatment extraoral photographs showed a convex lateral facial profile. The convex facial profile results from maxillary prognathism combined with mandibular retrognathia. The nasolabial angle measured ~90°, and the labiomental fold was shallow. The frontal view revealed a relatively longer lower third face height and facial asymmetry, with the chin point deviated 3 mm to the left. The lip and mentalis muscle hypertonicity was observed during lip closure. Although complete smiling photographs were not obtained, clinical examination confirmed the absence of a gummy smile (Figure 1).

The pretreatment intraoral photographs showed bilateral Angle Class I canine and molar relationships, but the last four molars on the right side exhibited a positive scissors bite. A deep overbite was observed in the #41 with right mandibular canine crossbite. The dental midlines of the upper and lower arches coincide with the facial midline. Maxillary and mandibular teeth had no gingival inflammation (Figure 1).

**Figure 2:**
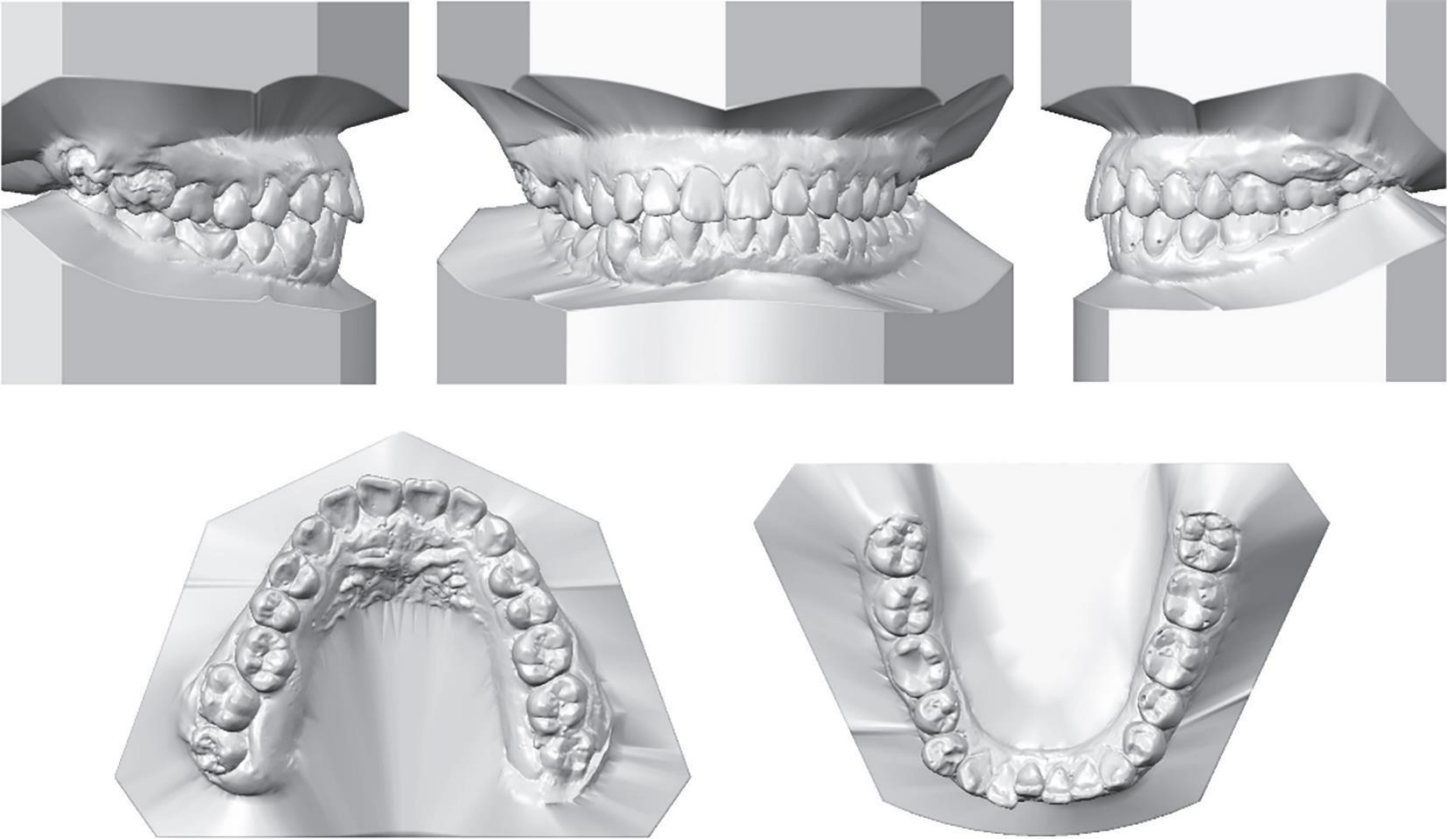
Pretreatment dental casts.

The dental casts before treatment showed no crowding in the maxillary arch and 3 mm of crowding in the mandibular arch. The overjet was 2 mm, the deep overbite in the #41 was 40%, and the severe deep curve of Spee was 3.5 mm. All permanent teeth up to the third permanent molars were present (Figure 2).

**Figure 3:**
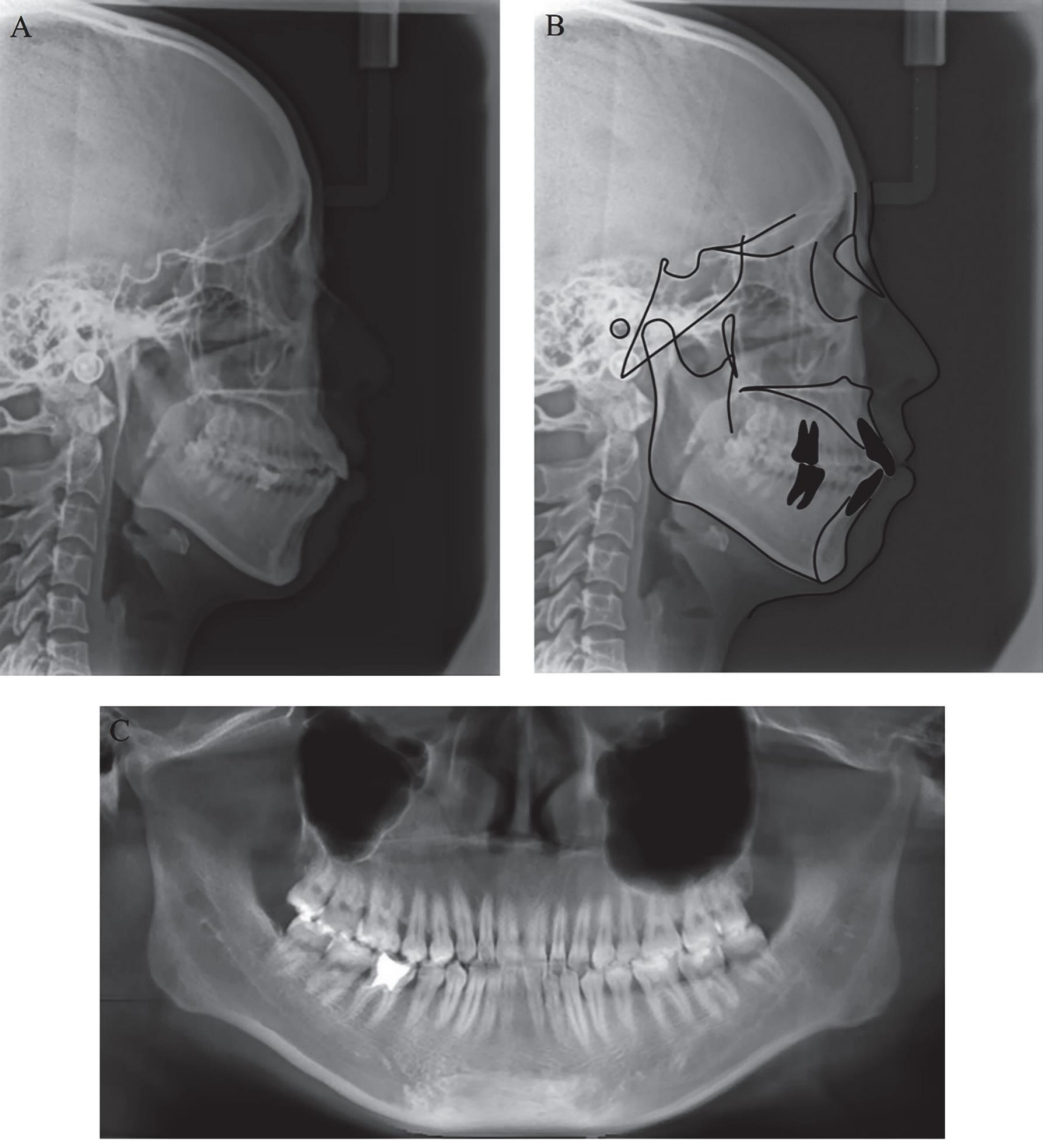
(A) Pretreatment cephalometric radiograph; (B) cephalometric tracing; and (C) panoramic radiograph.

The pretreatment lateral cephalogram and analysis showed a severe skeletal Class II jaw with a slight high plane angle of the mandible (ANB angle 6.7°, Wits appraisal 4.1 mm, MP–SN angle 39.7°). The maxillary incisors showed an average inclination (U1–NA angle 25.1° and U1–NA distance 5.0 mm), but the mandibular incisors were labially inclined (L1–NB angle 38.2° and L1–NB distance 11.0 mm), leading to the decrease of the interincisal angle (U1–L1 angle 110.0°). And the lower lip was protruding (*Z*‐angle 62.2°, LL–EP angle 3.6°). The panoramic radiograph pretreatment showed that the roots of the left maxillary molars were located at the bottom of the maxillary sinus. And the root canal treatment of the right first molar in the mandibule is not perfect (Figure 3; Table 1).

**Table 1:** Cephalometric measurements.

Measurement	Normal mean ± SD	Pretreament	Posttreament
SNA (°)	82.8 ± 4.0	85.3	83.8
SNB (°)	80.1 ± 3.9	78.6	78.8
ANB (°)	2.7 ± 2.0	6.7	5.0
NA–PA	6.0 ± 4.0	15.5	9.7
Wits (mm)	0 ± 2.0	4.1	−2
U1–NA (°)	22.8 ± 5.0	25.1	16.9
U1–NA (mm)	5.1 ± 2.4	5.0	1.7
L1–NB (°)	30.3 ± 5.8	38.2	26.2
L1–NB (mm)	6.7 ± 2.1	11.0	6.0
U1–L1 (°)	125.4 ± 7.9	110.0	133.1
U1–SN(°)	105.7 ± 6.3	110.4	99.2
L1–MP (°)	92.6 ± 7.0	99.6	87.6
MP–SN (°)	32.5 ± 5.2	39.7	39.9
MP–FH (°)	31.1 ± 5.6	29.5	29.7
ANS–Me/N–Me	55.4 ± 2.3	55.6	56.3
*Z*‐angle (°)	77 ± 5.0	62.2	83.6
LL–EP (mm)	1 ± 2	3.6	−2.0
UL–EP (mm)	−1 ± 1	−2.0	−4.3

In consequence, the patient was diagnosed with a convex facial profile, hyperdivergent skeletal Class II malocclusion, individual anterior crossbite, posterior scissor bite, and mild crowding in the mandibular dentition.

### 2.2. Treatment Objectives

The main dental and profile treatment objectives for this patient were to (1) correct the convex facial profile for esthetic enhancement, (2) align the maxillary and mandibular dental arches and level the curve of Spee, (3) correct the individual anterior crossbite and posterior scissor bite, and (4) achieve ideal intermaxillary incisor overbite and overjet relationships with good intercuspation.

### 2.3. Treatment Alternatives

To achieve these objectives, two possible treatment options were discussed with the patient. All treatment options required the extraction of the right third molars to correct the scissor bite. The first option was to correct the skeletal class II protrusion with a combination of orthognathic surgery and orthodontics. The bilateral mandibular first premolars were extracted preoperatively to establish anterior deep overjet. Le Fort I osteotomy with maxillary setback and bilateral sagittal split osteotomy (BSSO) for mandibular advancement combined with a genioplasty to improve esthetic profile. This treatment option would achieve a rapid and good result but with high risk and treatment costs.

The second treatment option involved extraction of all four first premolars, combined with miniscrew‐assisted absolute anchorage to maximize anterior teeth retraction. This approach can improve the convex facial profile while camouflaging skeletal Class II malocclusion.

After case discussion and risk‐benefit analysis, the patient decided to choose the second treatment plan and consented to the camouflage treatment alternative.

### 2.4. Treatment Progress

Before orthodontic treatment started, the patient underwent a 5‐month preparatory phase involving extraction of all four first premolars and two right third molars, endodontic retreatment of the mandibular right first molar, and periodontal scaling.

**Figure 4:**
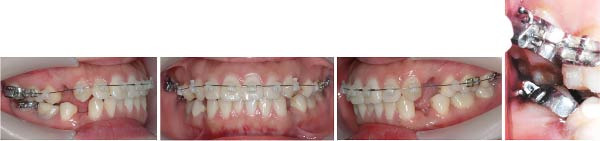
Fixed appliances were placed in the maxilla with a 0.012‐in Ni–Ti thermally activated arch wire, and the right second molars were drawn interactively with an elastic rubber band (~0.5 N), between which glass ionomer cement was used.

**Figure 5:**
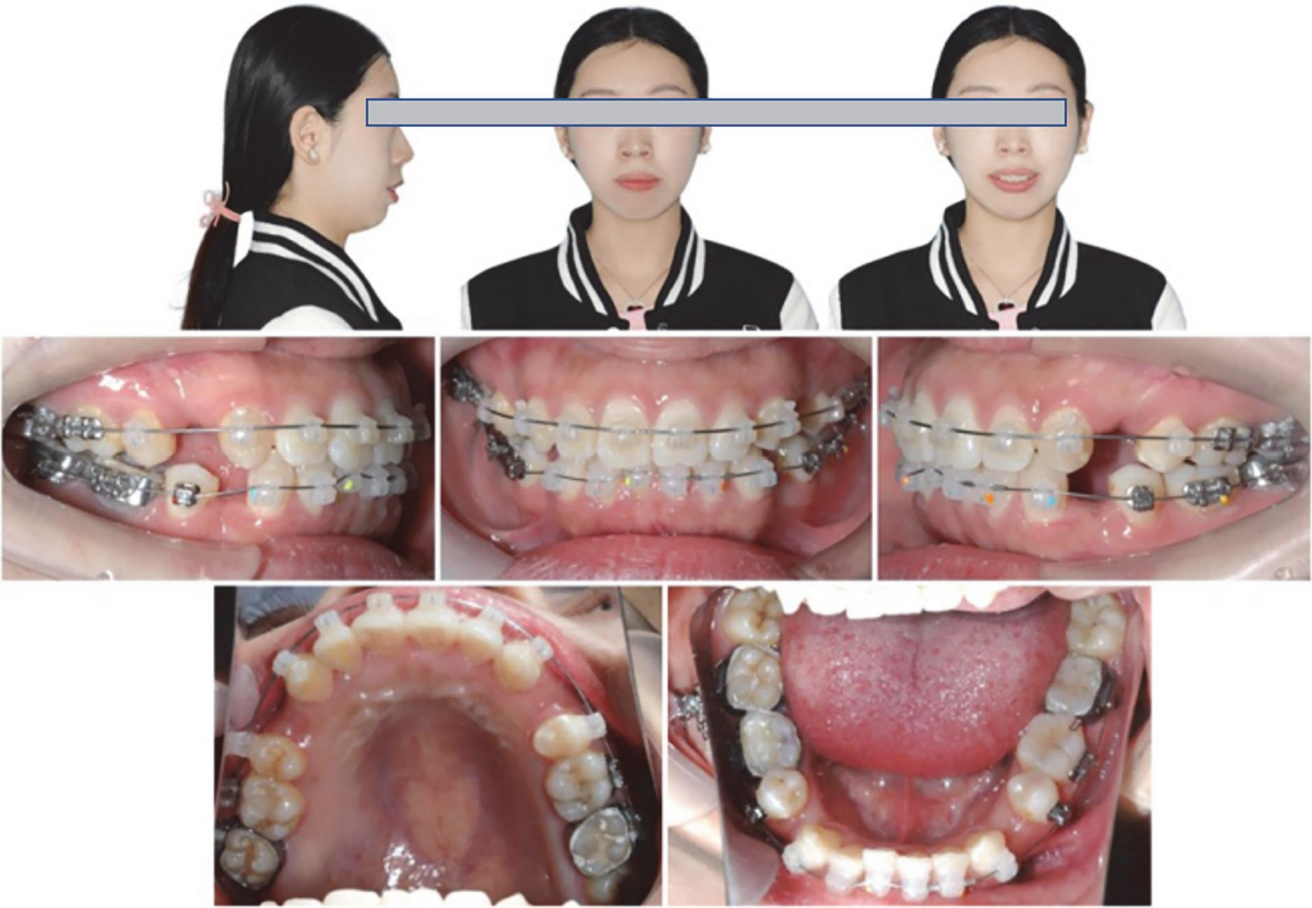
Two months after bonding, 0.014‐in Ni–Ti thermally activated arch wires were placed in both arches, and interactive traction was the same as before (~0.5 N).

Fixed ceramic self‐ligating appliances (Ormco, California, USA) were placed in the maxilla with a 0.012‐in Ni–Ti thermally activated arch wire, and the right second molars were drawn interactively with elastic rubber bands, between which glass ionomer cement was used to unlock the occlusion (Figure 4). Two months after bonding, 0.014‐in Ni–Ti thermally activated arch wires were placed in both arches, and interactive traction is the same as before (Figure 5).

Nine months after bonding, arch wires were adjusted to rectangular 0.019 × 0.025‐in stainless steel arch wires, and four 1.5 × 10 mm miniscrews (SYNTEC, Taiwan, China) were inserted into the alveolar bone between the second premolar and first molar on buccal sides of both arches. Two miniscrews in the maxilla combined with 10 mm long traction hooks were used to en masse retract the maxillary anterior teeth; two miniscrews in the mandible combined with 3 mm short traction hooks were used to retract and intrude the mandibular anterior teeth. The force was ~0.5 N.

**Figure 6:**
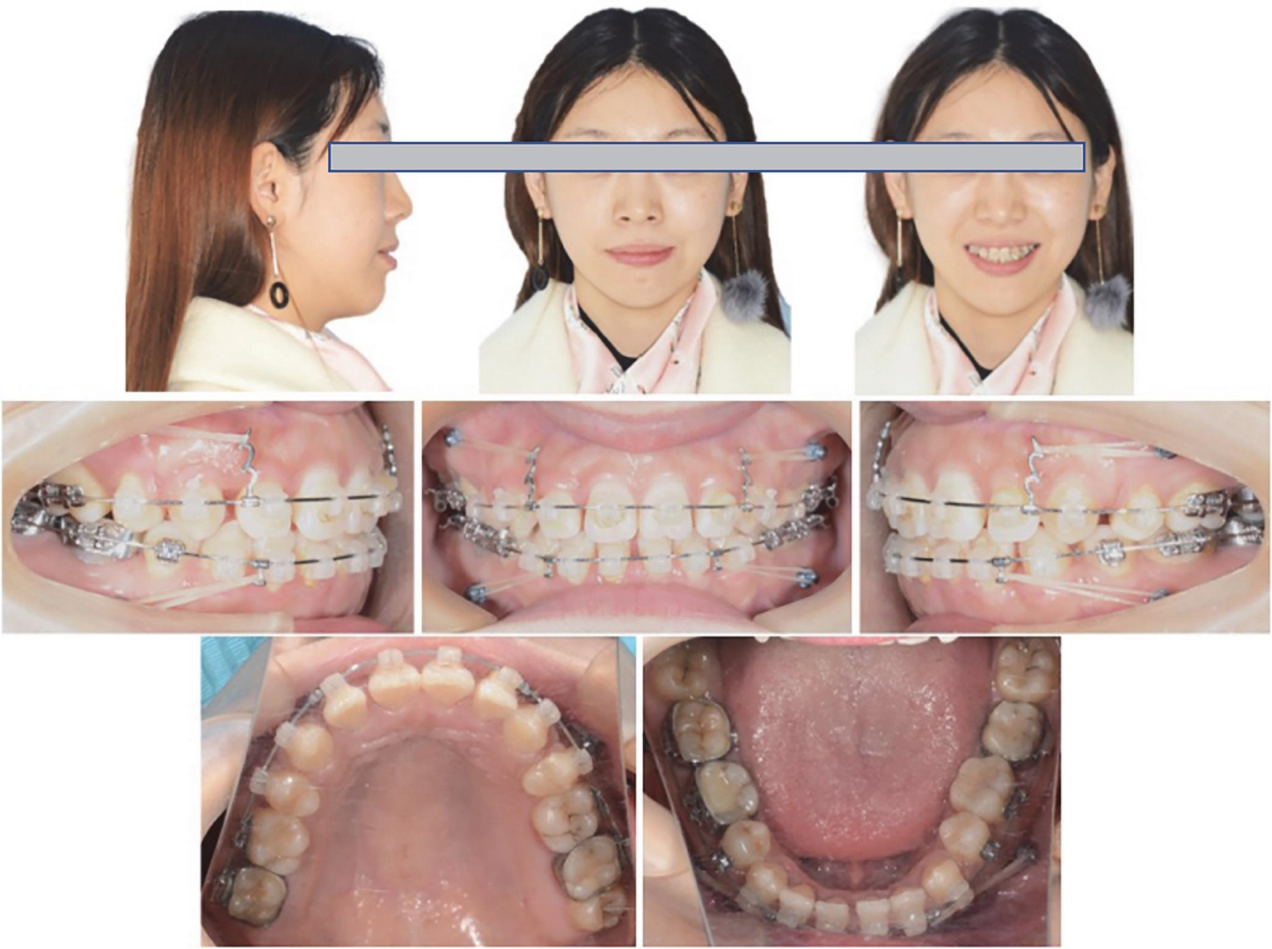
Twenty months after bonding, the arch wires were rectangular 0.019 × 0.025‐in stainless steel. The extraction space has significantly reduced with notable improvement in the patient’s facial profile.

**Figure 7:**
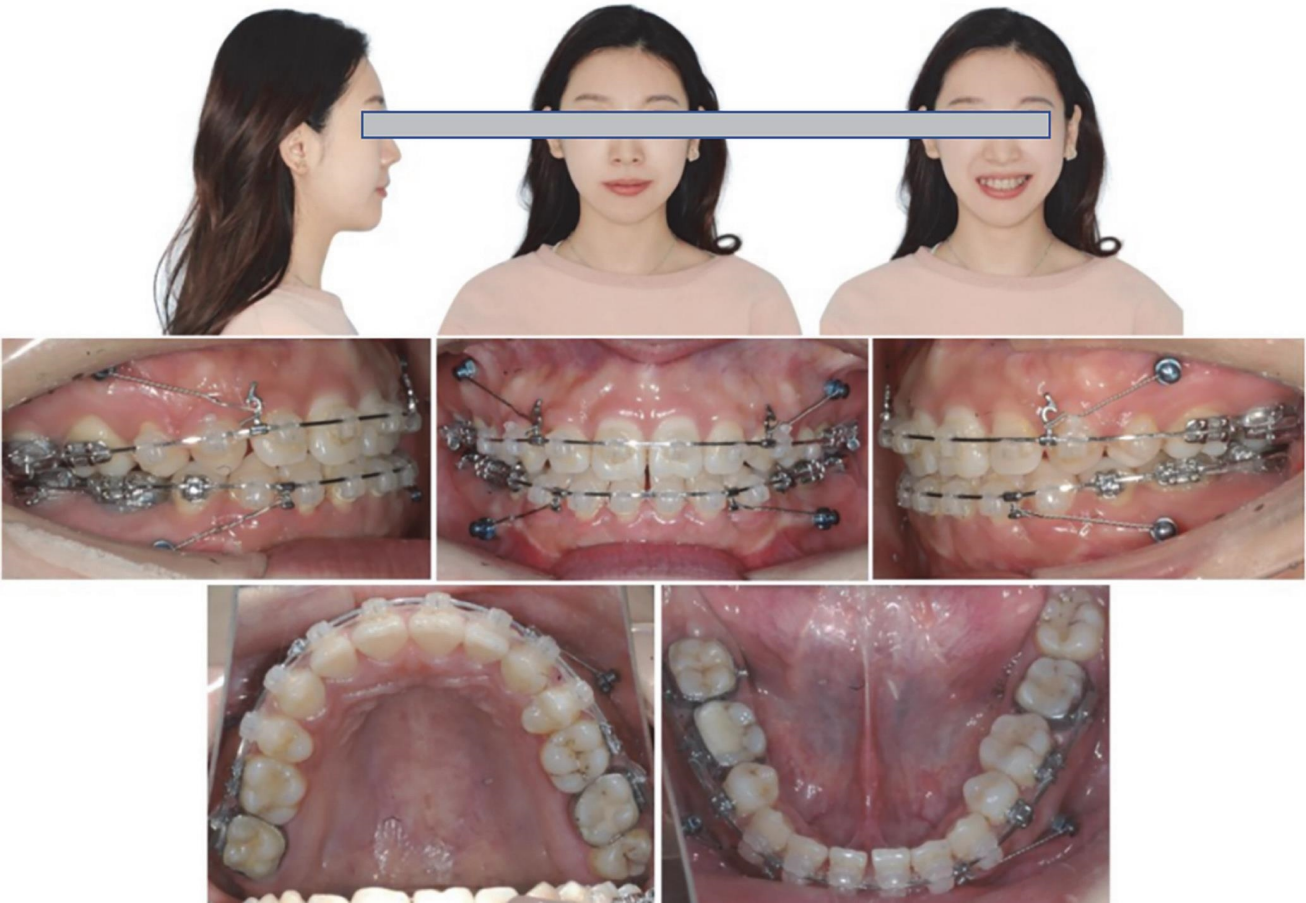
Twenty‐three months after bonding, the arch wires were rectangular 0.019 × 0.025‐in stainless steel. The Class I occlusion was achieved, little space was left, and 3 mm short traction hooks were used to retract and intrude the maxilla incisors for two months (~0.5 N).

**Figure 8:**
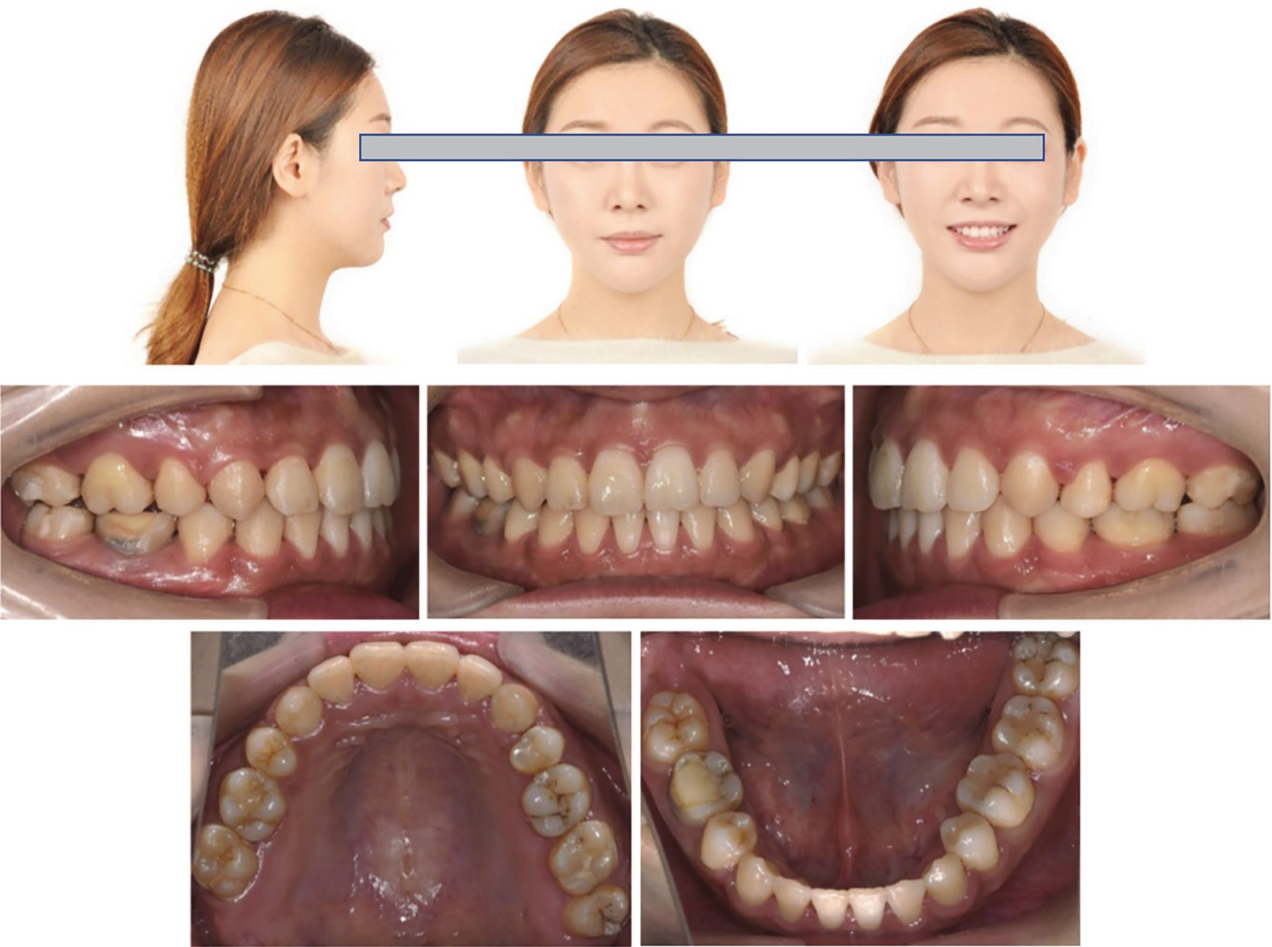
Posttreatment facial and intraoral photographs showed improved facial profile, ideal intercuspation, and normalized overjet and overbite.

Twenty months after bonding, the arch wires were rectangular 0.019 × 0.025 in stainless steel. The extraction space has significantly reduced with notable improvement in the patient’s facial profile (Figure 6). Twenty‐three months after bonding, the arch wires were rectangular 0.019 × 0.025 in stainless steel, the Class I occlusion was achieved, only a little space was left, but the patient was not so satisfied with her facial profile. Thus, 3 mm short traction hooks were used to retract and intrude the maxillary incisors for 2 months (Figure 7). After 6 months of arch coordination and precise occlusion adjustment, the fixed appliances and miniscrews were removed (Figure 8).

After a total of 31 months of active treatment, ideal overbite and overjet were achieved, a Class I molar relationship was realized, and the convex profile was improved significantly. After removing the miniscrews and appliances, the maxillary and mandibular teeth were then stabilized with clear thermoplastic retainers.

### 2.5. Treatment Results

**Figure 9:**
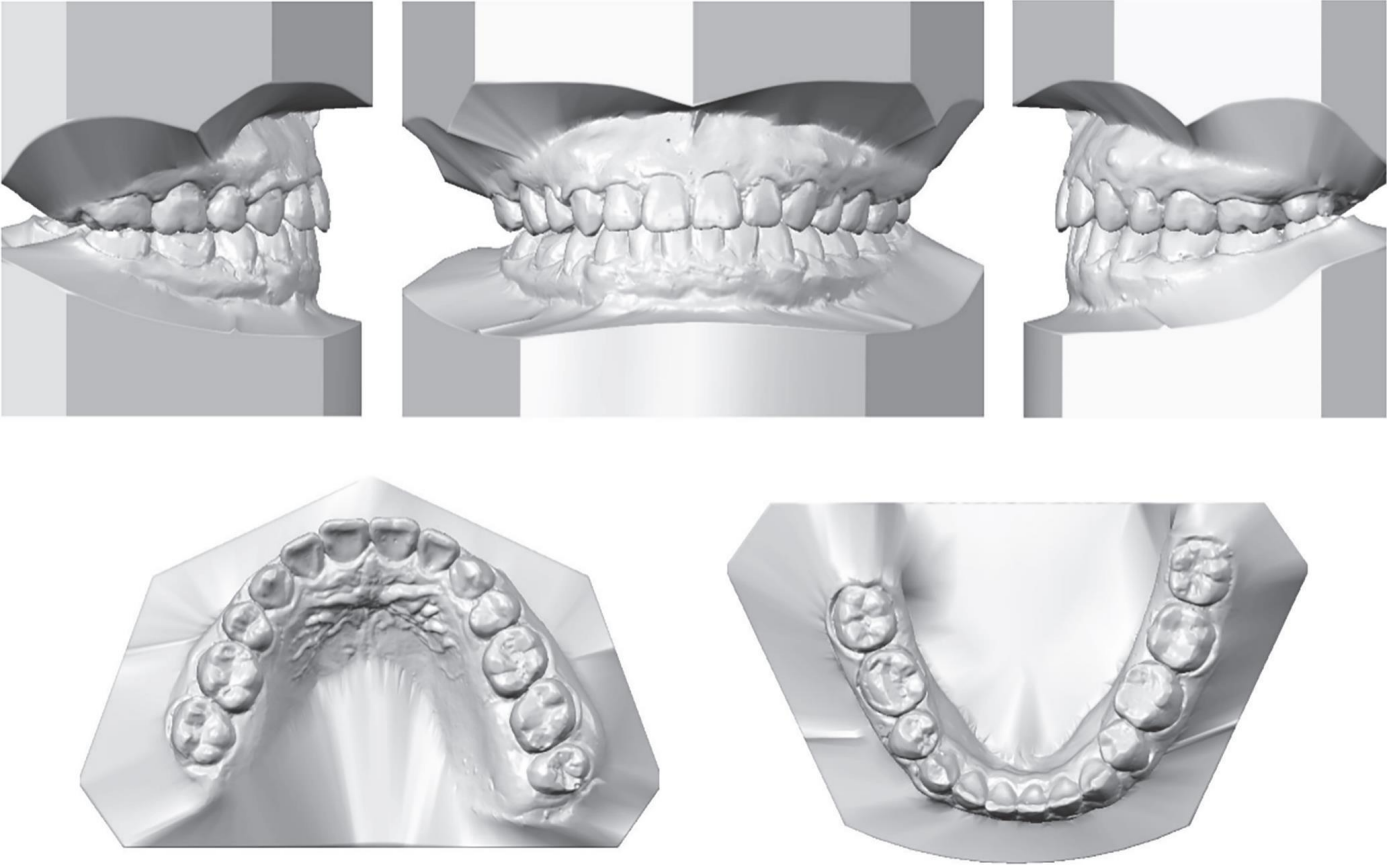
Posttreatment dental casts displayed well‐aligned dentitions, ideal intercuspation, and solid lingual occlusion.

After orthodontic treatment, the facial photographs showed that the protrusion of the facial profile was significantly improved, and the chin point was coincident with the facial midline. The lip and mentalis muscle were in normal tension during lip closure (Figure 8). Intraoral photographs and dental casts showed that the teeth were well aligned, the overbite and overjet were normal, the relationship between canines and molars was Class I, and the intercuspation was ideal (Figures 8 and 9). These changes were confirmed by the results of cephalometric analysis and cast superimpositions below.

**Figure 10:**
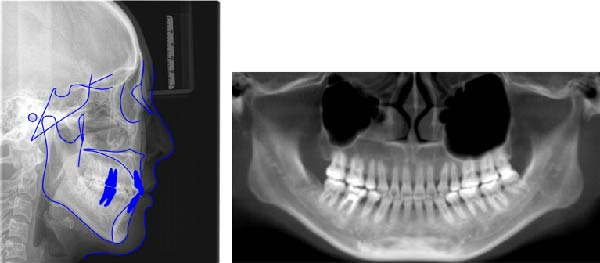
Posttreatment cephalograph, tracing, and panoramic radiograph.

The lateral cephalogram and cephalometric analysis showed that the upper alveolar seat point A moved backward and the facial convexity decreased (ANB angle 5°, decreased by 1.7°; NA–PA angle 9.7°, decreased by 5.8°); the mandibular plane angle and anterior height slightly increased (MP–SN angle 39.9°, increased by 0.2°; ANS–Me/N–Me ratio 56.3%, increased by 0.7%); and the labial inclination of the incisors decreased (U1–NA angle 16.9°, decreased by 8.2°; U1–NA distance 1.7 mm, decreased by 3.3 mm; L1–NB distance 6.0 mm, decreased by 5.0 mm), resulting in the interincisor angle increasing (U1–L1 angle 133.1°, increased by 23.1°); and the protrusion of the upper and lower lips decreased significantly (*Z*‐angle 83.6°, increased by 21.4°; the LL–EP distance was −2.0 mm, decreased by 5.6 mm, decreased by −4.3 mm, and decreased by 2.3 mm). The posttreatment panoramic film showed that the root canal retreatment of the right mandibular first molar was perfect; the extraction space was closed, the root parallelism was good, no obvious root resorption was observed, and no significant change in alveolar bone height was detected (Figure 10; Table 1).

**Figure 13:**
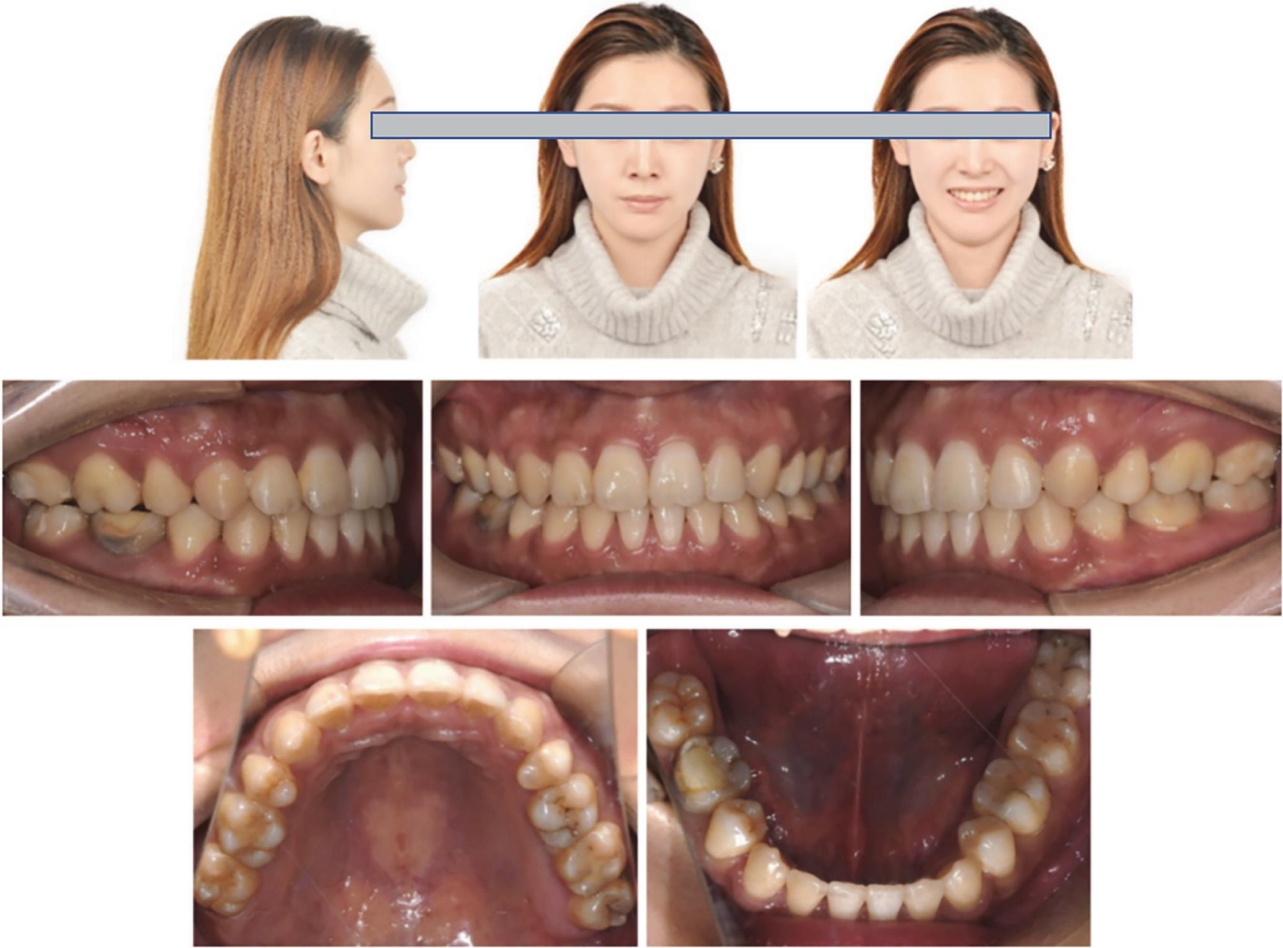
Twelve months after debonding, no obvious recurrence was found in the occlusion relationship and facial profile.

**Figure 14:**
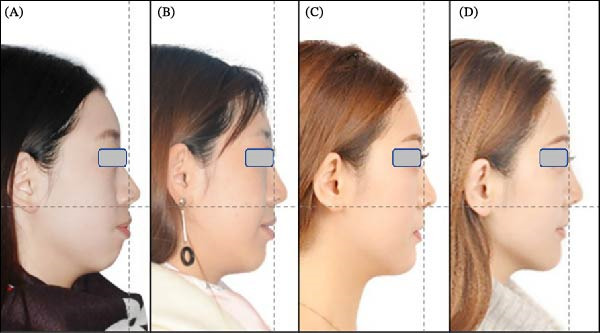
Facial profile transformation sequence: (A) pretreatment; (B) midtreatment (20th month); (C) posttreatment; and (D) one‐year postretention.

The cephalometric superimpositions showed that the incisors were retracted significantly, the facial profile was improved apparently, and the height of the posterior teeth remained unchanged (Figure 11). The dental cast superimpositions showed that the retraction of both arches was obvious, especially the anterior teeth (Figure 12). After 12 months of follow‐up, the occlusion relationship was stable, no obvious recurrence was found, and no significant change in facial profile was detected (Figures 13 and 14).

Figure 11Cephalometric superimpositions showed significant differences between pretreatment (black) and posttreatment (blue): (A) SN plane; (B) maxillary plane; (C) mandibular plane.

**Figure:**
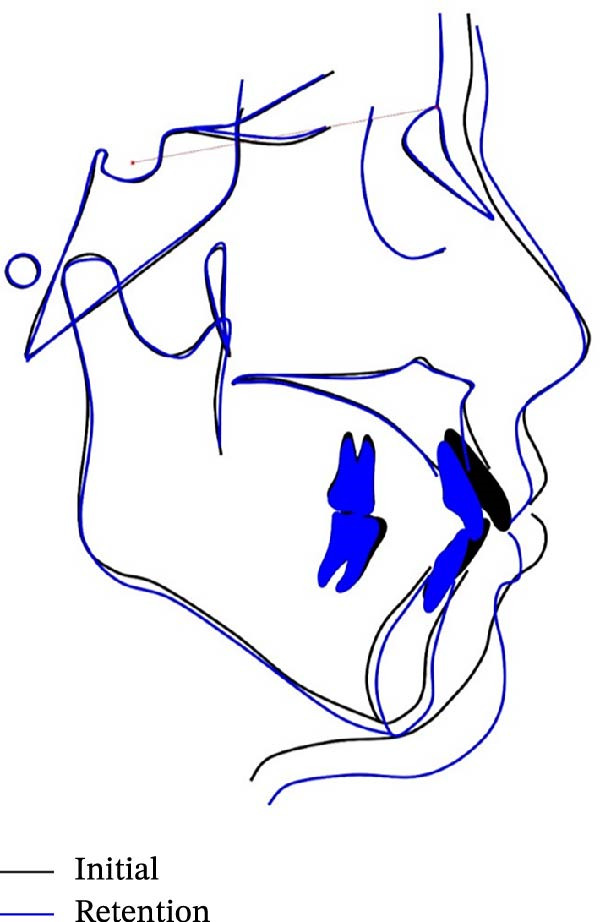
(A)

**Figure:**
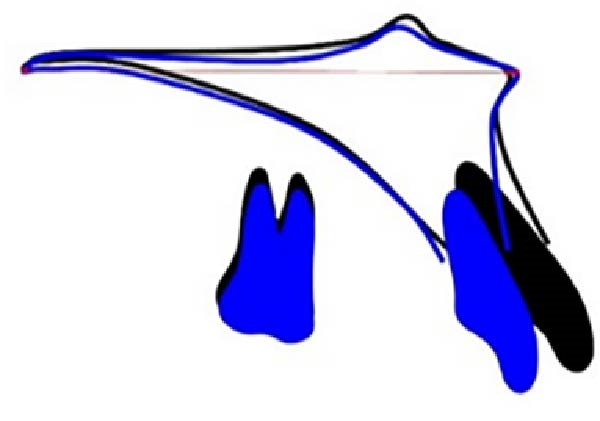
(B)

**Figure:**
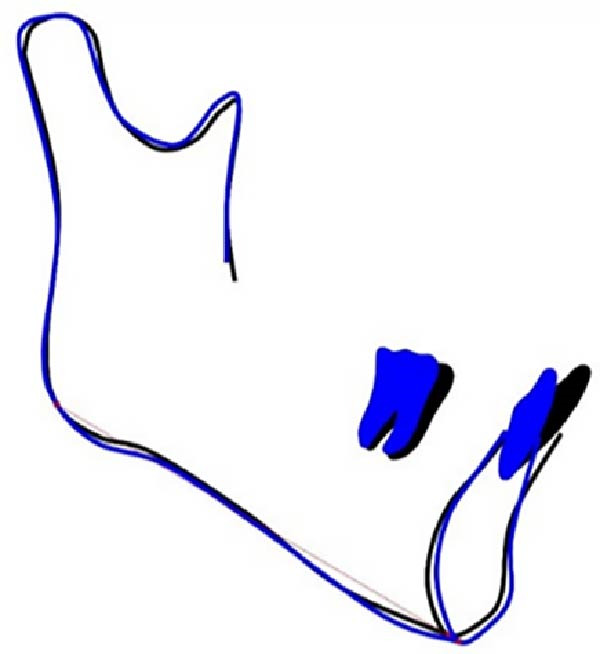
(C)

Figure 12Dental casts superimpositions showed obvious retraction of both arches, especially the anterior teeth.

**Figure:**
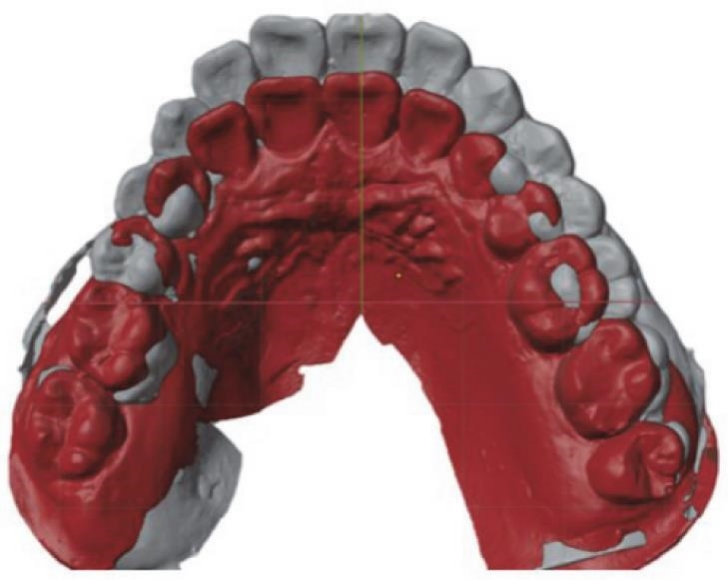
(A)

**Figure:**
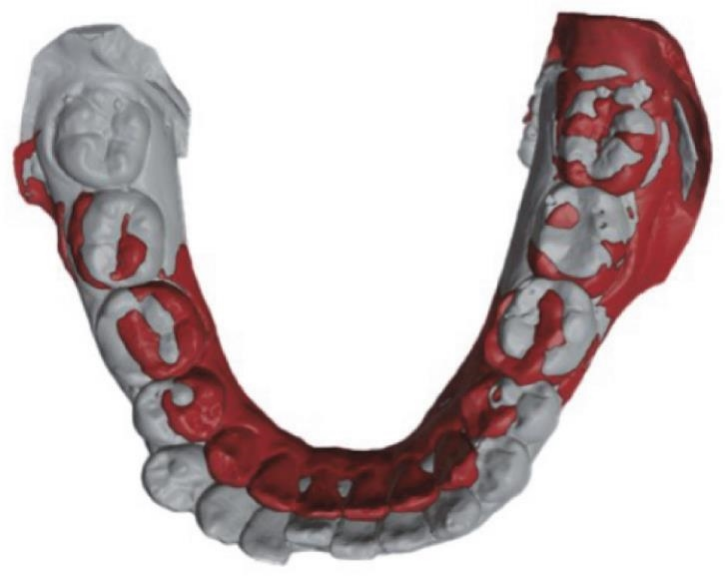
(B)

## 3. Discussion

Age and growth potential determine the treatment options for skeletal Class II malocclusion [5]. For patients with a skeletal Class II malocclusion who are still in the growth period, mandibular advancement should be considered as the primary treatment approach for the correction of skeletal Class II malocclusion with mandibular deficiency [17]. However, treatment of severe skeletal Class II malocclusion in adults remains a major orthodontic challenge. For adults with severe high‐angle skeletal Class II malocclusion, combined orthodontic‐orthognathic treatment remains the treatment of choice [18]. However, many patients decline surgical intervention due to concerns over operative risks and potential complications. Through detailed case analysis, orthodontic camouflage treatment may be considered as a viable alternative for select patients. The success of orthodontic camouflage treatment depended on precise and adequate anchorage control. Clinically conventional anchorage devices include headgear, lingual arches, transpalatal arches, and Nance holding arches [14]. Nonetheless, these anchorage devices present many limitations, particularly their dependence on patient compliance [19]. Currently, miniscrew anchorage has gained increasing clinical adoption. The miniscrew requires minimal patient cooperation and offers low invasiveness, high comfort, and cost‐effectiveness, with simple insertion and immediate loading capacity, which makes it the ideal anchorage device [20]. Studies demonstrated that miniscrew anchorage offers distinct advantages over conventional orthodontic appliances in treating Class II, division 1 malocclusion [14, 21]. The main symptom of the patient was the protrusion of incisors and lips, which was mainly caused by maxillary protrusion and mandibular retraction (SNA and SNB angles were close to the critical value). The case was managed by first premolar extractions utilizing miniscrews to maximize anterior segment retraction for the improvement of facial profile. Miniscrews are routinely implanted in the attached gingiva between the second premolar and first molar to achieve anterior teeth retraction [22]. In the process of the treatment, the key point is to effectively control the torque of the upper anterior teeth. By extending the traction hooks, the retraction force vector was aligned as closely as possible to the resistance center of the maxillary anterior teeth to optimize torque control. Nevertheless, the final outcome demonstrated excessive uprighting of the anterior teeth (U1–NA angle = 16.9°, U1–NA distance = 1.7 mm). This was directly attributable to the persistent demand of the patient for additional retraction to improve the facial profile during the late treatment stage.

For orthodontic camouflage treatment, it is necessary to control not only the sagittal movement of teeth but also the vertical movement. Studies demonstrate that miniscrews implanted in the maxillary posterior buccal region exhibit vertical control capacity, intruding maxillary molars and inducing mandibular counterclockwise rotation to improve facial profiles in Angle Class II patients [23, 24]. In this case, the patient exhibits a high mandibular plane angle (MP–FH = 29.5°, MP–SN = 39.7°). We aimed to induce counterclockwise mandibular rotation through maxillary molar intrusion. However, the root apices of the left maxillary molars were positioned at the floor of the maxillary sinus. A systematic review indicates that when tooth roots protrude into the maxillary sinus, apical root resorption may occur during tooth movement across the sinus floor [25]. Consequently, we avoided maxillary posterior intrusion, long‐distance molar movement, or Class II elastics, focusing solely on vertical molar height maintenance. However, the application of cross elastics to correct scissor bite during the initial treatment phase resulted in a minor increase in the mandibular plane angle (MP–FH = 29.7°, MP–SN = 39.9°), which did not compromise the facial profile enhancement.

During the application of miniscrew anchorage, in addition to the risks of miniscrew fracture and adjacent tissue damage during implantation, particular attention must be paid to potential complications, including root resorption of anterior teeth and palatal alveolar bone fenestration during significant retraction and intrusion movements. According to Liou and Chang [26], a comparative study evaluating miniscrew anchorage versus conventional anchorage in extraction cases demonstrated that while miniscrew anchorage allows for more maxillary en‐masse anterior retraction in patients with severe Class II cases, this advantage is accompanied by a significantly higher incidence of anterior tooth root resorption. Ahn et al. [27] evaluated the morphometric changes in the alveolar bone and roots of the maxillary anterior teeth after en‐masse retraction with maximum anchorage, and the results indicated that alveolar bone area and vertical bone level on the palatal side and root length and root area of incisors were significantly decreased. Barros et al. [28] conducted a comparative analysis of root resorption severity between anchorage modalities, demonstrating that most of the miniscrew anchorage group exhibited moderate to severe resorption, while most of the traditional anchorage group exhibited mild to moderate resorption. Therefore, orthodontic force must be strictly controlled within the 0.5–1 N range to mitigate iatrogenic root resorption during anterior tooth movement. In this case, the orthodontic force applied for anterior teeth retraction and intrusion was ~0.5 N (Figures 6 and 7). The panoramic radiograph revealed no obvious root resorption post‐orthodontic treatment (Figure 10). Furthermore, emerging cone‐beam computed tomography (CBCT) studies highlight the incisive canal as a potential limiting factor in maxillary central incisor displacement. Through CBCT analysis comparing central incisor relationships with the incisive canal before and after miniscrew‐assisted retraction, Pan and Chen [29] demonstrated that when central incisors are positioned more apically, their roots may contact the incisive canal during retraction movements, potentially leading to external apical root resorption. In this case, CBCT analysis revealed that the root apex of the right maxillary central incisors was positioned ~5 mm anterior to the incisive canal prior to treatment. Posttreatment evaluation showed a reduction in this distance, with no radiographic evidence of root encroachment into the canal (Figure 15).

Figure 15The distance from the right maxillary central incisor to the incisive canal: (A) pretreatment; (B) posttreatment.

**Figure:**
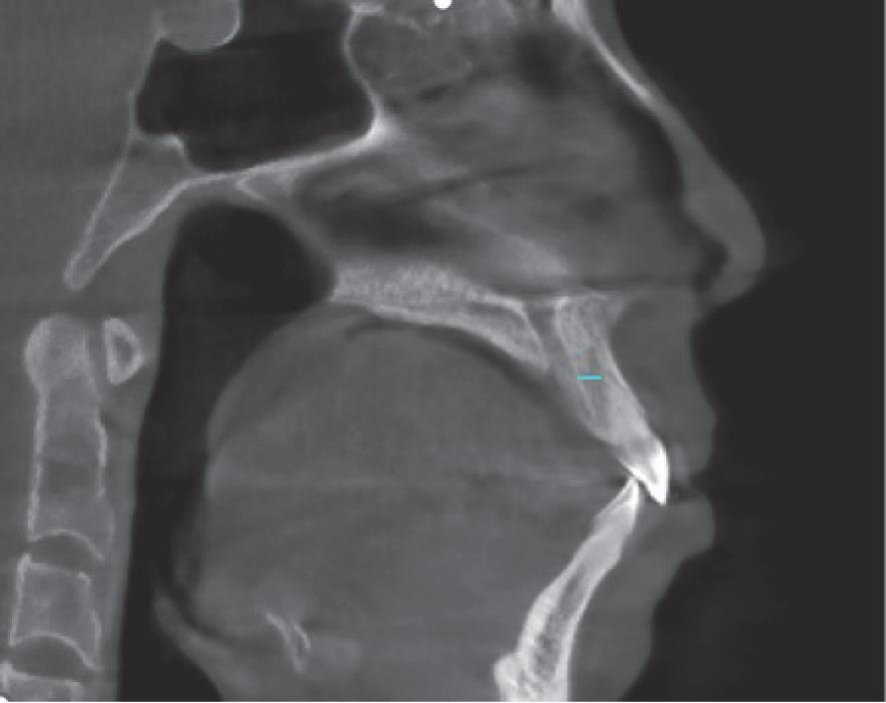
(A)

**Figure:**
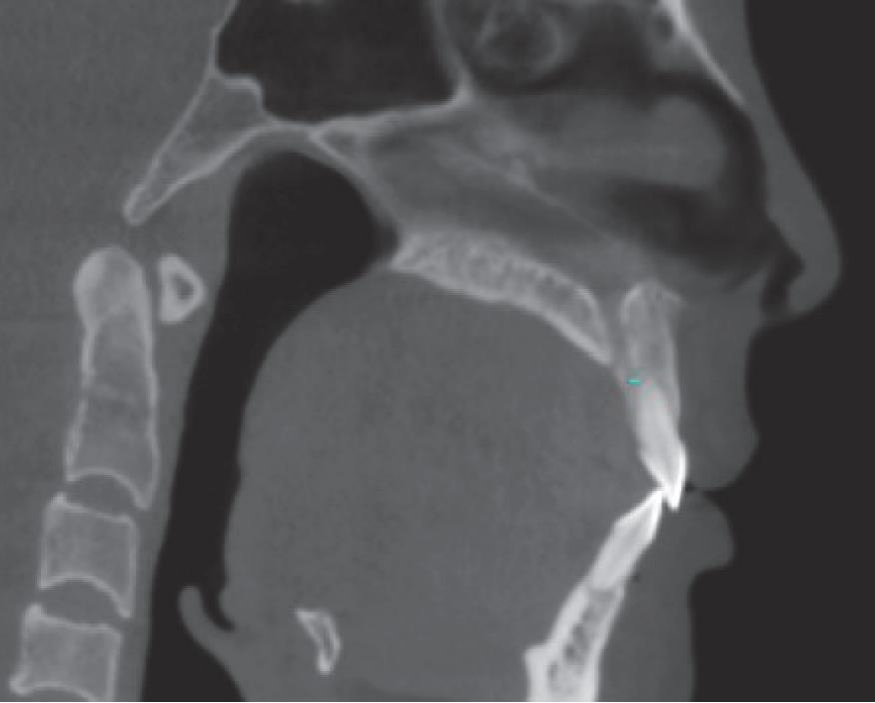
(B)

The patient was engaged in an art‐related profession and placed high importance on lip projection, with elevated demands for facial esthetics. Throughout the treatment process, we consistently prioritized facial harmony and maintained close communication with the patient, ultimately achieving esthetic goals. The chin morphology was also improved without deviation. The patient presented with a skeletal Class II malocclusion, labial inclination of mandibular anterior teeth, mentalis muscle strain during lip closure, and a shallow mentolabial sulcus. Posttreatment, the lower anterior teeth were retracted, with restoration of normal morphology and tension in the lip and mentalis muscles. The Pog point advanced significantly, and the *Z*‐angle showed marked improvement (increasing from 63° pretreatment to 83.6° posttreatment).

After a 12‐month retention period, no significant recurrence was observed. The facial profile and intraoral condition were well maintained, attributable to neuromuscular adaptation, proper soft tissue reorganization, and optimal cusp‐fossa occlusion. Masticatory and lip muscle functional training is particularly crucial for retention in high‐angle skeletal Class II malocclusion patients, especially those with short upper lip morphology. This patient exhibits slight upper lip insufficiency. Therefore, we requested daily muscle functional training, which may promote upper lip length augmentation and help maintain optimal facial profile. Most critically, patient compliance and awareness of the risk of recurrence must be established. The effects of various retention methods need to be further studied. For miniscrew‐assisted camouflage orthodontic treatment of hyperdivergent Class II malocclusion, extended follow‐up periods are required to demonstrate long‐term stability in occlusal relationship and facial profile.

## Funding

No funding was received for this manuscript.

## Consent

Informed consent was obtained from the patient in accordance with ethical principles, granting permission for the use and publication of their medical data, images, and case details in this report. The patient’s information has been anonymized to protect privacy and is intended for educational and research purposes.

## Conflicts of Interest

The authors declare no conflicts of interest.

## Data Availability

All data are contained within the article.
